# What should a learning health system look like?

**DOI:** 10.1136/bmjoq-2025-003455

**Published:** 2025-08-22

**Authors:** Robbie Foy, Paul Carder, Stella Johnson, Bethan Copsey, Sarah Alderson

**Affiliations:** 1 University of Leeds, Leeds Institute of Health Sciences, Leeds, UK; 2 NHS West Yorkshire Integrated Care Board, Bradford, UK; 3 Core Team - Research, West Yorkshire Research and Development, Wakefield, UK; 4 Leeds Institute of Clinical Trials Research, University of Leeds Faculty of Medicine and Health, Leeds, UK; 5 Academic Unit of Primary Care, University of Leeds, Leeds, UK

**Keywords:** Quality improvement, Randomised controlled trial, PRIMARY CARE

 Learning health systems have been defined as “a team, provider or group of providers in the health and care system that, working with a community of stakeholders, has developed the ability to learn from its own delivery of routine care and improve as a result”.^1^ The concept of learning health systems is gaining traction,^2^,^4^ including as a means of accelerating the translation of clinical evidence into practice. But how can healthcare system leaders and researchers ensure that their development moves beyond aspirations and rhetoric?

We draw on the experience of our collaborative evolution towards a primary care learning health system and consider the conditions necessary for such a system. We call for greater integration of research and quality improvement and a sharper definition of learning health systems.

## Barriers to the implementation of clinical evidence in primary care

Clinical research can only benefit patient and population health if findings are incorporated into routine care. There are delays and inappropriate variations in the uptake of evidence-based care and withdrawal of low-value or even harmful treatments.^5^ This translation gap limits the health, social and economic impacts of clinical research. Persistent inappropriate variations in care undermine efforts to achieve equity of outcomes; their magnitude cannot be explained away by population and casemix factors.

Primary care presents particular implementation challenges. In the UK, these include growing demand, increasing complexity of care and limited workforce capacity, against a background of recurrent organisational reconfigurations. There are also multiple competing priorities for attention, such as a steady stream of new guideline recommendations and quality indicators for performance management.^6^

Active strategies are needed to promote effective, efficient and equitable primary care. Addressing deficits in knowledge and resources is important, but insufficient by itself to bring about significant change.^7^ There is a substantial and growing evidence base to inform implementation strategies. For example, rigorous evaluations of interventions such as audit and feedback, computerised decision support and local opinion leaders all demonstrate improvements in patient care and outcomes.^8^

However, there are pitfalls in applying this evidence base to improvement efforts. First, it is hard to predict with confidence whether a given implementation strategy will work for a given targeted evidence-based practice. Some degree of judgement and acceptance of risk is inevitably required. Second, the effectiveness of most implementation strategies is typically modest, although still potentially important at a population level. This is partly because new randomised trials often test implementation interventions against control (no intervention) conditions rather than actively exploring how to enhance effectiveness through head-to-head trials of different interventions; this contributes to research waste.^9^ Third, there is limited evidence on the cost-effectiveness of implementation strategies and uncertainty about which targeted priorities would yield the greatest returns on investment, thereby handicapping decision-making in the face of competing priorities.

## Integrating research and quality improvement

Our academic-commissioner collaboration has started to address these barriers to implementation. We began with an agreement that the academic team would lead bids for competitive grant income focused on implementation research while the primary care commissioner (responsible for planning and purchasing services to improve population healthcare and outcomes) would act as the host National Health Service (NHS) organisation for research and actively support governance and delivery. The commissioner also provided pump-priming funding for posts to augment bidding capacity. Despite successive commissioner reconfigurations, there has been continuity in the personnel supporting research functions, now based within one NHS Integrated Care Board for West Yorkshire.

**Figure 1:**
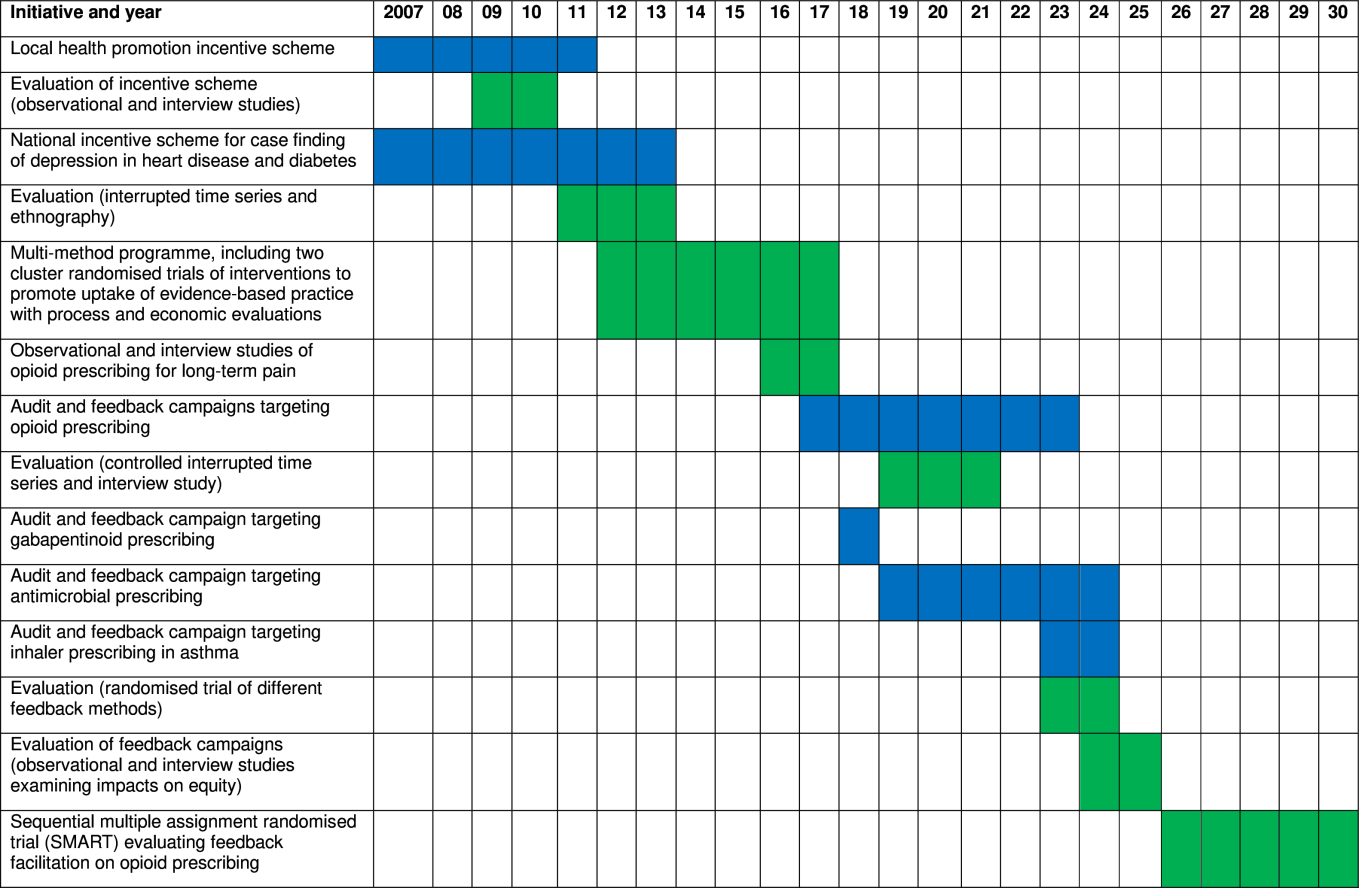
Timeline summarising sequencing and evolution of quality improvement activities (in blue) and evaluations (in green).

Our grant-funded research began with evaluations of local and national incentive schemes targeting primary care.^10 11^ We progressed to highly pragmatic (‘real-world’) and efficient randomised trials assessing the cost-effectiveness of a multi-faceted intervention involving audit and feedback on the implementation of evidence-based indicators, which demonstrated cost-effectiveness in targeting high-risk prescribing (figure 1).^12^ Around the same time, we identified a concerning rise in potentially harmful opioid prescribing for chronic, non-cancer pain in primary care.^13^ With support from our NHS commissioners, we jointly established and rolled out an audit and feedback campaign targeting opioid prescribing across over 300 general practices in West Yorkshire. A controlled interrupted time series analysis identified a 5.6% relative reduction in opioid prescribing, equivalent to 15 000 fewer people prescribed opioids, a net saving of £700 000 in prescribing costs and an estimated 10-fold return on investment.^14^ The biggest reduction was in people aged over 75, who are at higher risk of opioid-related falls and death, and there was no compensatory rise in the prescribing of other analgesics or referrals to musculoskeletal services. The campaign was subsequently rolled out and reached over 1000 practices across Yorkshire and the Humber and North East England and has been recognised in NHS guidance on maximising the benefits of research.^15^

Following the success of the opioid prescribing campaign, we progressed to further audit and feedback campaigns in response to NHS priorities. These targeted reductions in low-value prescribing (gabapentinoids for chronic pain and antimicrobials) and promoted more effective and greener prescribing of asthma inhalers. These campaigns have been rolled out to other regions and continue in response to local demand. Qualitative studies suggested high acceptability by general practices, especially when the campaigns promoted clinically relevant and achievable goals.^16^ As interviews with practice staff suggested that hard copy, paper feedback reports might be preferred in addition to emailed feedback, we designed and conducted an efficient trial to compare their effects.^17^

Our efforts to align quality improvement and research continue. These include an evaluation of whether feedback campaigns reduce inequalities in over-prescribing^18^ and a Sequential Multiple-Assignment Randomised Trial design to optimise feedback to reduce prescribing of opioids for chronic non-cancer pain.^19^

## Learning health systems and their potential value

Recognising the extent of gaps between recommended and actual clinical practice, a 2006 US Institute of Medicine roundtable first proposed the concept of a ‘learning healthcare system’.^20^ While this concept has since evolved and spread, most literature in this field is still largely theoretical rather than empirical.^21^

Learning health systems offer a vehicle for optimising implementation strategies and building a cumulative science. This includes the design and delivery of head-to-head trials of interventions. As well as adding to knowledge on how to enhance effectiveness, such trials can appeal to healthcare commissioners and providers as no participating practices are denied interventions. For example, a primary care organisation with an established audit and feedback programme may wish to establish whether adding practice facilitation to feedback makes a worthwhile difference. Sometimes there is a strong underpinning evidence base for such changes. However, there is often uncertainty around whether any changes have resulted in desired impacts. Examining trends over time is useful, but attributing any improvements or declines in performance to programme changes can be challenging, given the complex contexts of and multiple other influences on healthcare delivery (pandemics being an extreme example of a system disruption). This uncertainty about effectiveness is not only an ‘academic’ concern. Time and money spent on additional interventions such as practice facilitation will be wasted if they do not improve care. Randomised trials provide robust evidence of effects, but setting up and delivering individual trials can be costly and time-consuming in the absence of an established infrastructure.

Learning health systems can combine repeated cycles of data-driven improvement with robust evaluation. When they incorporate randomised or rigorous quasi-experimental evaluations, learning health systems produce reliable evidence of effectiveness. By virtue of being embedded within existing large-scale programmes using routinely collected data, they can be relatively efficient while delivering real-world evidence. Learning health systems represent a shift from researchers coming into healthcare systems to ‘do improvement and evaluation’ towards greater collaboration that directly addresses organisational needs and delivers research sustainably embedded within systems rather than becoming an ornamental burden.

## What conditions are necessary for a learning health system?

A growing range of frameworks is now available to guide efforts to move towards learning health systems. For example, Reid *et al* recognise the need for five capabilities: advanced analytics and population insights; evidence synthesis and curation; patient, caregiver and provider co-design; implementation and reach; and rapid cycle evaluation, feedback and adaptation.^4^ We suggest several conditions necessary to establish a learning health system based on our experience and earlier work (box 1).^22^

Box 1 **Suggested conditions for a learning health system**

**Leadership and organisation**

Mutual stability to promote continuity in the partnership between healthcare system and researchers.

**Values and expectations**

A shared understanding of equipoise to ensure that ‘negative’ evaluation results are not misrepresented as research failures or a lack of impact of improvement activities.

General practice trust in the data and improvement methods.

**Priority setting**

Systematic selection of clinical priorities for change so that they are underpinned by a strong evidence base, offer scope for improvement and can provide sufficient returns on investment.

**Resources and logistics**

Availability of data to assess performance and processes or resources for improvement.

Sufficient methodological skill mix in the core team with access to wider experience and skills as needed.

An ‘engine house’ to design candidate interventions which draws on practical knowledge, empirical evidence and theoretical perspectives.

Aligned resources and timelines for intervention delivery (eg, compiling and disseminating performance feedback) and evaluation (eg, design and analysis).

Stable programme funding to allow long-term planning and system evolution.

**Governance and monitoring**

Regular contact to monitor and troubleshoot improvement and evaluation activities.

Proportionate approaches to ethical oversight and governance arrangements that balance protections and research burdens for individuals and organisations.

Demonstration of benefits to healthcare system and patient populations.

Mutual organisational stability is required to promote continuity in partnerships between healthcare systems and researchers and foster a shared vision. Our partnership has matured from being transactional (ie, dependent on mutual favours) towards becoming transformational (with genuinely shared goals). Our long-term aim is to develop a self-sustaining system which combines continuous improvement with rigorous evaluation. However, it takes time to build improvement activities and evaluations of increasing ambition. Although we are approaching two decades of collaboration, there are continuing threats to the sustainability of our learning health system, such as the uncertainties involved in securing competitive grant funding, changes in personnel and further health service reconfigurations. Our current work combines external funding from a series of short to medium-term research grants with NHS staff resourcing. Both of these are vulnerable to changing funding climates or changes in organisational leadership and priorities.

While learning health systems can drive systematic improvement, a shared understanding of the nature of experimentation is necessary to ensure that any ‘negative’ evaluation results are not misrepresented as failures. It is as useful to know what does not work as well as what does. Furthermore, a trial demonstrating that a new implementation strategy is not more effective than an existing strategy may take place against an underlying trend of improvement.

We advise expectation management and guarding against unrealistic ambitions; an early learning health system such as ours cannot solve all of the challenges facing primary care, but it can make more efficient use of limited resources for improvement, including the time and energy of practice staff. General practices working at the ‘sharp end’ of improvement may be unaware of higher-level system goals but do need to trust the data enough to act on feedback and engage with other improvement methods.^23^

Clinical priorities for change should be systematically selected so that they are underpinned by a strong evidence base, offer scope for improvement and can provide sufficient returns on investment. Our work to date has been largely responsive; a more strategic approach could involve integrating health economics throughout recurrent evaluation cycles, that is, beyond a standard cost-effectiveness evaluation plugged into the end of a trial. For example, there are competing priorities for implementation and evaluation in primary care; are the greatest gains likely to be achieved through targeting diabetes, asthma or depression care and is this most likely to be cost-effective through audit and feedback, educational webinars or computerised decision support? Modelling of different scenarios may indicate which targets and interventions are (1) highly likely to be cost-effective, and hence worth implementation rather than evaluating, (2) promising but uncertain, and hence worth evaluating and (3) highly unlikely to be cost-effective, and hence not worth pursuing. This approach may help further reduce research waste while guiding ongoing implementation strategies.

The availability of data to assess performance and improvement infrastructure (eg, for audit and feedback) is fundamental. There are opportunities to develop learning health systems within other data-driven improvement programmes. For example, we have delivered randomised trials with parallel process and economic evaluations evaluating different ways of enhancing the effects of feedback in partnership with national clinical audit programmes.^24 25^

The core team should possess a sufficient skill mix, including quality improvement, data management and analysis and implementation science, with scope to bring in other experience and skills as needed, such as topic-specific clinical leadership and qualitative methods. This means that any programme of work can take a holistic approach. While randomised trials and rigorous quasi-experiments (such as interrupted time series analyses) can answer questions about effectiveness, other parallel studies can inform delivery and generate knowledge. Process evaluations, often using qualitative methods of enquiry, can offer important insights into why interventions work or do not. Economic evaluations can inform decisions about whether any benefits outweigh intervention costs.

The identification, development and application of interventions should be based on existing evidence and theory as well as available resources and skills. For example, our programme of work to date has focused on audit and feedback. This has a well-established evidence base that includes methods of amplifying effects, such as incorporating actionable plans with specific advice for improvement.^26^ Intervention design can also draw on theoretical perspectives, such as embedding behaviour change techniques within feedback reports.^27^ We further draw on our collective experience in data collection and analysis and feedback design in delivering and scaling up our feedback campaigns. However, there are opportunities to develop and test further implementation strategies with established evidence bases and associated uncertainties about how to maximise effects, such as computerised clinical decision support systems.^28^

It can be challenging to align both resources and timelines for improvement activities and evaluation. For example, research and commissioning partners inevitably share risks in seeking competitive grant funding for evaluations and improvement activities that may need to be postponed to align with grant funding cycles and set-up processes for research. Therefore, stable programme funding can substantially facilitate long-term planning and system evolution.

Regular formal and informal contact between collaborators is essential to maintain relationships and to anticipate and solve problems. We have worked closely together to promote proportionate approaches to ethical oversight and governance that balance protections and research burdens for individuals and organisations. For example, our aforementioned randomised trials evaluating the effects of a multi-faceted intervention on the implementation of evidence-based indicators used an ‘opt-out’ approach to recruiting general practices.^12^ Practices were included in the trials unless they actively declined participation. As well as minimising administrative burden for practices and facilitating the attainment of recruitment targets, this approach also meant that our findings were more likely to be applicable to most general practices than those which had expressed an interest in research.

The demonstration of tangible benefits for the healthcare system, such as specific improvements in population healthcare, and for the research partners, such as grant income and academic outputs, can help drive the virtuous cycle underpinning the partnership. Our work demonstrating a reduction in opioid prescribing contributed to demands for further feedback campaigns. More broadly, healthcare systems and health funders under pressure to deliver more within limited resources may welcome a whole-system approach to estimating returns on investment of the learning health system. This would need to identify, for example, all of the operational costs (eg, staff time) involved in establishing and running the learning health system as well as any wider benefits to healthcare delivery and population health. The latter might include improved patient outcomes associated with organisational participation in research.^29^

## A learning health system: are we there yet?

In writing this article, we initially debated among ourselves about whether we could legitimately claim to have established a learning system. Some examples elsewhere appear to resemble quality improvement collaboratives or communities of practice,^1^ while others incorporate robust effectiveness evaluations.^30^ The latter approach offers the advantage of both driving improvement and delivering generalisable knowledge. Whatever operational definition is preferred, we would now agree that a learning health system ‘is an ongoing journey rather than a destination’”.^2^

We have established a partnership that integrates research and improvement in a drive for more effective, efficient and equitable care. Evidence of our evolution includes shifts from separate to shared goals, from being either researcher or service-driven to collaboratively owned, from being project-focused to programmatic and progression with varying levels of scale, methodological rigour and novelty.

There is a myth that there are necessary trade-offs between real-world relevance and robust research. There need not be any compromise. Rigorous effectiveness evaluations can be integrated within healthcare systems and large-scale improvement programmes, an approach that was advocated within the original conceptualisation of learning health systems.^20^ In recognising future learning health systems, we propose that such features are definitional rather than optional.
